# A comparative analysis of three distinct rehydrating solutions for addressing liver tissue desiccation resulting from dehydration failure

**DOI:** 10.7717/peerj.21363

**Published:** 2026-05-27

**Authors:** Weiping Shi, Guangming Fu, Hailei Shi, Huiyang Qi, Haiyang Fu

**Affiliations:** Department of Pathology, The Affiliated Hospital of Qingdao University, Qingdao, Shandong, China

**Keywords:** Rehydrating, Sandison’s solution, Formol-Glycerol solution, Gordon’s solution, Tissue dehydration

## Abstract

**Background:**

The tissue dehydration process is an important step in pathology specimen preparation. Dehydration failure, caused by equipment malfunction, incorrect program settings, or operator error, is a recognized problem in laboratory practice and can compromise the quality of hematoxylin and eosin (H&E) slides, thereby hindering subsequent pathological diagnosis. Common *ad hoc* responses include stepwise rehydration or short-term immersion in various buffers. However, information on repairing over-desiccated specimens is limited, and there is little systematic evidence comparing different rehydration approaches. We evaluated three rehydration solutions for restoring tissue morphology and H&E staining quality after over-desiccation, using standardized morphological scoring to inform evidence-based guidance for handling of dehydration incidents in diagnostic histopathology.

**Methods:**

Three common rehydrating solutions were compared. After a 24-hour period of routine fixation by 10% neutral buffered formalin, the liver specimens were manually dehydrated with anhydrous ethanol and left to air dry for eight hours. The specimens were then immersed in three distinct rehydrating solutions for either 12 hours or 24 hours. After the rehydration, dehydration, and embedding processes, the specimens were sectioned and stained. Then, they were observed under a microscope, and a quantitative assessment of nuclear morphology was performed to evaluate the recovery effects of the three repair solutions.

**Results:**

The dried liver tissues were restored in all specimens soaked in one of the three repair solutions, with the 24-hour group demonstrating superior results compared to the 12-hour group. Gordon’s solution exhibited the most pronounced repair effect, while Sandison’s solution and Formol-Glycerol solution also demonstrated restorative effects, albeit with residual issues.

**Discussion:**

The results indicated that, regardless of restorative solution used, the tissues exhibited some degree of recovery; however, the restoration of their morphology effects was not the same. The 24-hour group showed a superior level of tissue repair compared to the 12-hour group. Of the three solutions, Gordon’s solution after 24-hour immersion was the most effective at promoting tissue repair.

## Introduction

Tissue dehydration constitutes a critical component within the broader framework of quality control in the domain of histopathology, as it is essential for producing high-quality paraffin blocks, which serve as a fundamental foundation for subsequent examinations. However, in the event of tissue dehydration failure, a multitude of complications may occur, including the following: tissue shrinkage; brittle tissue; interference with staining properties; distortion of tissue; dry, scratchy tissue that is difficult to cut during microtomy; and nuclear streaming (Chapman, 2019; Chatterjee, 2014; Rastogi et al., 2013; Satapute, Shashikala & Gu, 2020; Taqi et al., 2018). These complications are a significant source of diagnostic challenges, thereby impeding the accurate identification of lesions (Rastogi et al., 2013; Taqi et al., 2018).

Currently, the most frequently used equipment for tissue dehydration in pathology departments are closed tissue processors, although some open tissue dehydration processors also remain in use. The closed tissue processor offers a degree of protection by maintaining a closed environment during malfunctions, thereby preventing tissue exposure to air. In contrast, open processor has the potential to cause tissue stagnation in the air or environment, which can result in further damage. In our study, we encountered an issue that required resolution: a malfunction of the open tissue processor exposed tissues to the air after anhydrous ethanol treatment, causing them difficult to handle or manipulate. This study tests three rehydrating solutions and their ability to recondition dried liver tissues after tissue desiccation due to a malfunction in the dehydration process.

## Materials & Methods

### Materials

A total of 50 liver tissue specimens, each measuring 1.5 cm × 1.5 cm × 0.5 cm, were obtained and prepared for the experiment. This study was approved by the Institutional Review Board of the Medical Ethics Committee of the Affiliated Hospital of Qingdao University, China (QYFY WZLL 30317), and complied with the principles of the Declaration of Helsinki. All participants provided written informed consent prior to participation in the study. Three rehydrating solutions were created, as shown in Table 1. The rehydrating solutions were prepared and stored in sizable plastic specimen containers with leak-proof lids.

**Table 1:** Composition of rehydration solutions used in this study.

Solution	Components
Sandison’s solution	30 mL anhydrous ethanol 1% formaldehyde: 5 mL formaldehyde^a^ 45 mL tap water 5% sodium carbonate: 1 g sodium carbonate 20 mL tap water
Formol-Glycerol solution	10 mL formaldehyde^a^ 2 g sodium acetate 90 mL tap water 10 mL glycerol solution^b^
Gordon’s solution	50 mL anhydrous ethanol 20 mL tap water 30 mL glycerol solution^b^ 1 g sodium bisulphite

**Notes.**

a Formaldehyde was used as a 37% (w/v) stock solution.

b Glycerol was used as pure glycerol (100% v/v).

### Methods

1. Three rehydrating solutions were prepared according to the formulas described above, with sufficient volume to ensure complete immersion of the liver specimens.

2. After 24-hour fixation in 10% neutral buffered formalin, the liver tissues were divided into four groups for subsequent analysis. Group 1, fifteen specimens were dehydrated through a graded ethanol series to anhydrous ethanol, then air-dried for 8 h. Each specimen was then individually immersed in 50 mL of one of the three rehydrating solutions (*n* = 5 per solution) for 12 h. Group 2, fifteen specimens were dehydrated to anhydrous ethanol and air-dried as described above. Each specimen was subsequently individually immersed in 50 mL of one of the three rehydrating solutions (*n* = 5 per solution) for 24 h. Group 3, ten specimens were dehydrated to anhydrous ethanol and air-dried for 8 h, but were not subjected to any rehydration treatment. Following these treatments, the specimens were conventionally dehydrated, clearing, and embedded. Group 4, ten specimens as the control were processed using the standard dehydration protocol, which involved dehydration through a graded ethanol series without the intermediate air-drying step, and proceeded directly to clearing and embedding.

3. The paraffin blocks were routinely sectioned to a thickness of 3 µm. Coated or adhesive slides were used to prevent the detachment of tissue sections during the staining process.

4. After the preparation stage, the sections were stained with hematoxylin and eosin (H&E) for histological examination.

5. The primary evaluation criterion was the morphological recovery of hepatocytes following rehydration, especially the hepatocyte nuclei. To assess hepatocyte recovery with different rehydration solutions, two pathologists, blinded to the experimental groups, examined the HE-stained slides under a light microscope (Olympus BX41) at magnifications of 100x and 400x. The degree of hepatocyte recovery was assessed using a semi-quantitative scoring system (0 = no recovery, 1 = mild recovery, 2 = moderate recovery, 3 = significant recovery) based on morphological criteria of hepatocyte nuclear morphology. To ensure the reproducibility of the histological assessment, both pathologists independently scored all slides. For intra-observer reliability, each pathologist re-evaluated the same slides after a one-month washout period, with slides randomly re-ordered to minimize recall bias. Statistical analyses were performed using SPSS software (version 22.0, IBM Corp., Armonk, NY, USA). Inter-observer and intra-observer reliabilities were assessed using the intraclass correlation coefficient (ICC) based on a two-way mixed-effects model for absolute agreement, reporting single measures with 95% confidence intervals. A *p*-value < 0.05 was considered statistically significant.

6. Subsequently, to perform a quantitative assessment of nuclear morphology, the HE-stained images were analyzed using ImageJ software (Version 1.53t; National Institutes of Health, Bethesda, MD, USA). The mean cross-sectional area of the nuclei was measured in five random high-magnification (400 ×) fields per slide using the following protocol. First, the hematoxylin channel was isolated using the ‘Color Deconvolution’ plugin with the preset H&E vector. The resulting image was converted to 8-bit grayscale, and a threshold was applied to generate a binary mask of the nuclei. To separate adjacent nuclei, the ‘Watershed’ algorithm was applied. Finally, the ‘Analyze Particles’ function was used to measure the nuclear area, with a size exclusion filter (particles <500-pixel^2^ were excluded to eliminate non-nuclear debris). All measurements were expressed in pixel^2^, as all images were acquired at the same magnification (400 ×), allowing direct comparison between groups.

## Results

### Observer variability

To ensure the reproducibility of the histological assessment, inter-observer and intra-observer reliabilities were evaluated for hepatocyte recovery scores. The inter-observer intraclass correlation coefficient (ICC) was 0.82 (95% confidence interval (CI) [0.66–0.91], *P* < 0.001), indicating excellent agreement between the two pathologists (Table 1). Intra-observer reliability analysis revealed ICC values of 0.84 (95% CI [0.69–0.92]) for Observer A and 0.83 (95% CI [0.67–0.91]) for Observer B, demonstrating high repeatability for both observers (Table 2).

### Histological assessment

Having established excellent inter-observer agreement, we evaluated hepatocyte nuclear morphology as the primary criterion for recovery following rehydration.

**Figure 1:**
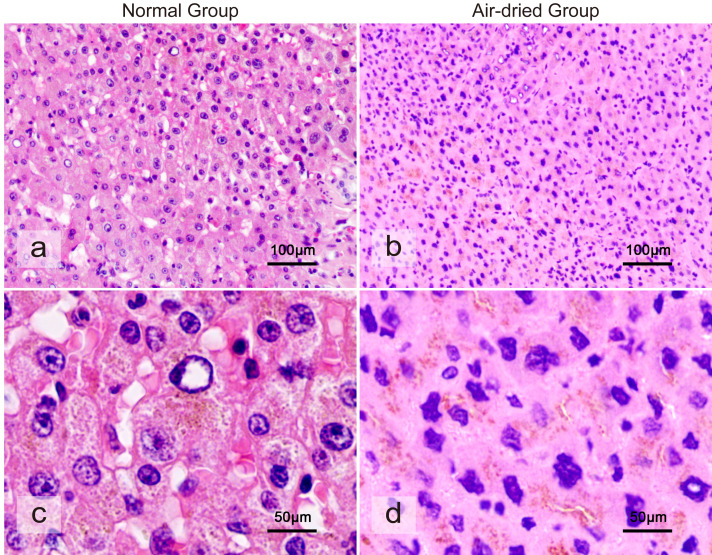
A morphological analysis of the differences between normal group and directly dehydrated liver tissue after air-dried. (A) The microscopic morphology of liver tissue following a standard dehydration process. (B) The microscopic morphology of liver tissue after direct dehydration following air-dried. (C) High-magnification morphology of normal liver tissue morphology. (D) High-magnification morphology of liver tissue after being subjected to direct dehydration following air-dried.

The liver tissue following a standard dehydration process offers a clear visual representation of its structural organization, and the nuclei, cell membranes, and interstitial spaces are distinctly discernible (Fig. 1). However, tissue specimens that were subjected to direct dehydration after drying became hard and produced the poor-quality paraffin blocks, resulting in poor-quality H&E slides (Fig. 1). Upon microscopic examination, liver tissues subjected to direct dehydration following drying exhibited clear signs of wrinkling, an indistinct cellular structure, significantly reduced interstitial substance, a substantial loss of tissue structure, and virtually undetectable morphology. The nuclei were contracted and densely distributed, and the cell membranes were nearly invisible.

**Table 2:** Inter-observer and intra-observer reliability for hepatocyte recovery scores.

Parameter	ICC	95% CI	*p*-value
**Inter-observer reliability**	0.82	0.66–0.91	<0.001
**Intra-observer reliability**			
Observer A	0.84	0.69–0.92	<0.001
Observer B	0.83	0.67–0.91	<0.001

**Notes.**

ICC intraclass correlation coefficient CI confidence interval

ICC was calculated using a twoway mixed-effects model for absolute agreement. Analyses were performed using SPSS version 22.0.

After rehydration treatment, the dried liver tissues were restored in all specimens immersed in one of the three repair solutions, and the tissue specimens were softer than those subjected to direct dehydration after drying. A visual examination of the samples revealed that the Gordon’s solution exhibited the most pronounced swelling of the dried liver tissue, followed by the Formol-Glycerol solution, with Sandison’s solution exhibiting the least swelling. Subsequent tactile evaluation confirmed that specimens treated with the Gordon’s solution were the softest, followed by those treated with Sandison’s solution, while specimens treated with Formol-Glycerol solution were the hardest, with the 24-hour group demonstrating superior results in comparison to the 12-hour group. Furthermore, for each solution, the 24-hour immersion group yielded superior tissue softness compared to 12-hour group.

**Figure 2:**
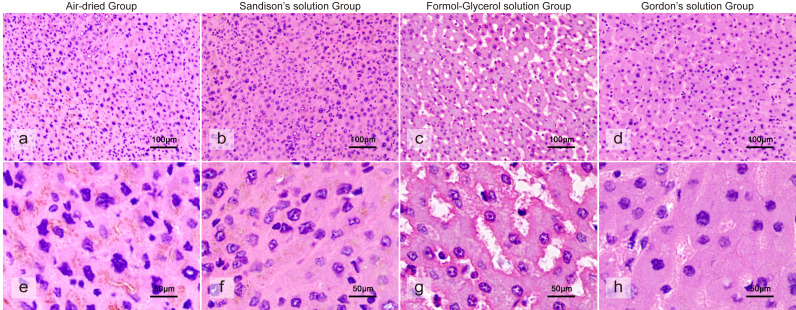
The histological morphology of liver tissues after a 12-hour immersion in one of three rehydration solutions. (A) Microscopic morphology of liver tissue after direct dehydration following air-dried. (B) Microscopic morphology of liver tissue after a 12-hour immersion in Sandison’s solution. (C) Microscopic morphology of liver tissue after a 12-hour immersion in Formol-Glycerol solution. (D) Microscopic morphology of liver tissue after a 12-hour immersion in Gordon’s solution. (E) High-magnification morphology of liver tissue after direct dehydration following desiccation. (F) High-magnification view of liver tissue after a 12-hour immersion in Sandison’s solution. (G) High-magnification view of liver tissue after a 12-hour immersion in Formol-Glycerol solution. (H) High-magnification view of liver tissue after a 12-hour immersion in Gordon’s solution.

Twelve hours after the repair process, the three solutions were compared. Although the tissues treated with all three solutions demonstrated restorative effects, they also have some difference in the extent of repair (Fig. 2). Sandison’s solution was found to be more effective at repairing cell nuclei. A visual analysis of the tissues reveals that, in comparison to desiccated tissue, the shrunken nuclei exhibited signs of swelling and repair, and the interstitial spaces appeared to be slightly expanded. However, the dense distribution of the nuclei showed no significant improvement. Some nuclei appeared swollen, while others remained shrunken, all exhibiting irregular shapes with invisible cell membranes. Formol-Glycerol solution demonstrated a restorative effect on tissues when compared to desiccated tissue. In the 12-hour Formol-Glycerol-treated tissue, the nuclei appeared enlarged and the stroma exhibited swelling, but numerous unidentified interstitial spaces were present throughout the tissue. Nuclear morphology was generally preserved, although condensation was still evident, and cell membranes remained invisible. In comparison with desiccated tissue, the 12-hour Gordon’s solution-treated tissue exhibited minimal improvement in tissue recovery. It exhibited the least effect on nuclear repair. While some nuclei exhibited indications of turgidity recovery, the majority remained in a state of solidification and contraction, with cell membranes barely perceptible. However substantial improvement was observed in interstitial recovery.

**Figure 3:**
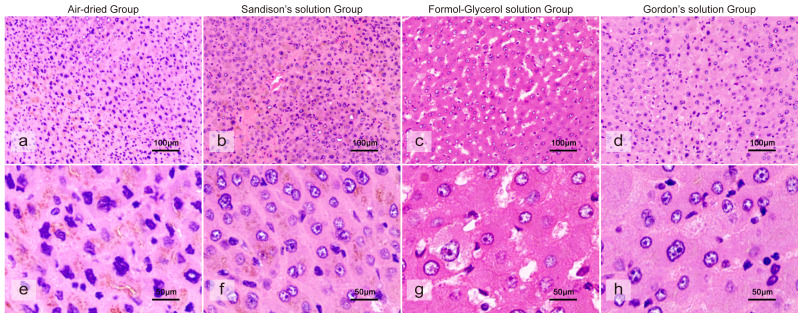
The histological morphology of liver tissue after 24-hour immersion in one of three rehydration solutions. (A) Microscopic morphology of liver tissue after direct dehydration following air-dried. (B) Microscopic morphology of liver tissue after a 24-hour immersion in Sandison’s solution. (C) Microscopic morphology of liver tissue after a 24-hour immersion in Formol-Glycerol solution. (D) Microscopic morphology of liver tissue after a 24-hour immersion in Gordon’s solution. (E) High-magnification morphology of liver tissue after direct dehydration following desiccation. (F) High-magnification view of liver tissue after a 24-hour immersion in Sandison’s solution. (G) High-magnification view of liver tissue after a 24-hour immersion in formol-glycerol solution. (H) High-magnification view of liver tissue after a 24-hour immersion in Gordon’s solution.

After twenty-four hours of repair, the cell nuclei in tissues treated with Sandison’s solution exhibited a more pronounced effect in comparison to those in the 12-hour group and those subjected to direct dehydration after drying (Fig. 3). Histological examination revealed enlarged cell nuclei throughout these tissues, displaying relatively regular shapes, with minimal improvement in the stroma. However, the dense distribution of the cell nuclei did not appear to improve, and the cell membranes in these tissues remained imperceptible. Tissues treated with Formol-Glycerol solution showed persistent swelling in the nuclei and interstitial swelling. Although cell nucleus recovery improved, the presence of unexplained spaces within the tissue remained pronounced in the 24-hour group. In these tissues, the cell membranes were also barely perceptible. Tissues treated with Gordon’s solution showed notable recovery of the cell nuclei, accompanied by a substantial increase in the number of repaired cell nuclei. A marked improvement in cell nuclei was observed, accompanied by parallel improvement in the stroma. In these tissues, the cell nuclei manifested uniform swelling and recovery, while some membrane structure became faintly recognizable.

Moreover, a comparative analysis of the three groups revealed that tissues treated with Gordon’s solution exhibited the optimal morphological recovery after a 24-hour immersion period (Fig. 3). Furthermore, the nuclei in tissues treated with Formol-Glycerol solution or Gordon’s solution exhibited a morphology closer to normal than those treated with Sandison’s solution. However, it was only Gordon’s solution that demonstrated a slight effect on the cell membrane; in contrast, the other two rehydrating solutions exerted no effect on either the cytoplasm or cell membrane of the tissues.

Except for hepatocytes, other hepatic cell types were still observed. No bile ducts or bile duct epithelial cells were definitively identified in any of the examined slides, they were suspected but not clearly evident, and fibroblasts were not prominent. Sinusoidal cells showed variable morphology by group, mainly in clarity and degree of dilation. In Sandison’s solution, sinusoidal cells were largely obscured at 12 h and only faintly discernible at 24 h with indistinct boundaries. In Formol-Glycerol solution, sinusoidal cells were apparent at both times but showed abnormal dilation and morphology. In Gordon’s solution, sinusoidal cells were indistinct at 12 h and showed only marginal improvement, remaining faint at 24 h. No large vessel profiles or clear vascular cells were identified.

### Quantitative analysis of nuclear area

To objectively validate these findings, nuclear area was measured using ImageJ software (Version 1.53t; National Institutes of Health, Bethesda, MD, USA).

Nuclear area measurements for all experimental groups are summarized in Table 3. One-way ANOVA revealed significant differences among the groups (*F* (7, 42) = 6.846, *P* < 0.001).

Compared to the Normal Group (3,089 ± 61.79-pixel^2^), most experimental groups exhibited significantly reduced nuclear areas (*P* < 0.001), indicating incomplete morphological restoration. However, the nuclei treated with Gordon’s solution after 24-hour immersion showed no significant difference from the Normal Group (*P* = 0.9482), suggesting that their nuclear morphology was restored to a level comparable to normal.

Compared to the Air-dried Group (1,850 ± 20.92-pixel^2^), all three rehydration solutions (Sandison’s, Formol-Glycerol, and Gordon’s solutions) resulted in significantly larger nuclear areas (all *P* < 0.001), confirming their reparative effects on nuclear repair.

Compared to the 12-hour Immersion Group, both Formol-Glycerol and Gordon’s solutions resulted in significantly larger nuclear areas after 24-hour immersion (both *P* < 0.001), whereas Sandison’s solution showed no significant difference (*P* = 0.9717). These findings suggest that extended immersion time facilitates nuclear recovery, but this effect is solution-dependent, with Sandison’s solution lacking the reparative capacity observed for the other two.

## Discussion

In this study, tissues were dehydrated to simulate tissue dryness caused by dehydrator malfunction. The dried tissues were then treated with three distinct rehydration solutions, and the differences in the repair effects of three rehydration solutions were subsequently examined.

Previous studies have attempted to repair desiccated tissues in a variety of ways. For example, one study (Toba, Sanders & Carreon, 2023) used a formaldehyde-glycerol solution to repair dried heart tissues, which resulted in a gradual restoration of the external morphology of the tissues. However, the authors did not carry out staining of the dehydrated tissues, so there is no firsthand information about the microscopic morphology of the tissues after repair. A separate study used Sandison’s solution for the repair and staining of post-mortem skin specimens, yielding a specific outcome: the procedure was found to be both useful and effective (Boracchi et al., 2016; Collini et al., 2014; Gentile et al., 2020). However, this investigation did not address the repair of tissue dryness caused by machine malfunction during routine operations.

**Table 3:** Nuclear area measurements and pairwise comparisons with reference groups. The *n* values in this table represent the total number of nuclei measured per group. Data are presented as means ± SD. *P*-values were calculated using one-way ANOVA with Tukey’s *post hoc* test for multiple comparisons. *P* < 0.05 was considered statistically significant.

	Normal group	Air-dried group	12-hour Immersion Group			24-hour Immersion Group		
			Sandison’s solution	Formol-Glycerol solution	Gordon’s solution	Sandison’s solution	Formol-Glycerol solution	Gordon’s solution
*n*	1,232	2,639	2,339	1,244	1,186	2,062	1,103	810
Nuclear area (pixel^2^)	3,089 ± 61.79	1,850 ± 20.92	2,656 ± 38.65	2,198 ± 47.36	2,362 ± 45.87	2,602 ± 43.50	2,599 ± 62.40	2,998 ± 67.73
*p*-value *vs* Normal Group		<0.0001	<0.0001	<0.0001	<0.0001	<0.0001	<0.0001	0.9482
*p*-value *vs* Air-dried Group			<0.0001	<0.0001	<0.0001	<0.0001	<0.0001	<0.0001
*p*-value *vs* 12-hour Group						0.9717	<0.0001	<0.0001

In the present study, all three rehydration solutions tested resulted in tissue swelling when compared to the dry state. This finding suggests that all three treatments exerted an influence on the tissue’s overall morphology. However, this assessment was based on visual inspection rather than precise dimensional measurements. When these tissues were observed under the microscope, the repair of the nucleus was most significant, with the nucleus being restored from a severely shrunken state to a more expanded state, consistent with the quantitative measurements of nuclear area. However, the performance of each rehydration solution was inconsistent, and none of the three repair fluids was able to help repair the cytoplasm or the cell membrane of the tissue. The nuclei of the tissue cells treated with Sandison’s solution demonstrated the most rapid recovery in morphology, and the distribution of these nuclei was found to be denser, irrespective of immersion time. It is hypothesized that reparative swelling of the tissues occurred following by Sandison’s solution, resulting in swelling of the nuclei and the interstitium. However, it is predicted that the interstitium undergoes a greater degree of retraction following rehydration, while the repair of the nuclei is maintained.

Formol-Glycerol solution has been demonstrated to facilitate the recovery of the nucleus, exhibiting a swelling effect on the tissue (Neisskenwirth, 2020; Tambuzzi et al., 2022; Van Rosendal et al., 2010). However, the presence of unidentified gaps in the tissue has also been observed, indicating that the underlying cause of the tissue damage may be multifactorial. One potential factor is the alkaline nature of the Formol-Glycerol solution. The pH of solution may have undergone a change after its interaction with the formaldehyde solution and sodium acetate, potentially creating an acidic or alkaline environment, similar to a Cannizzaro reaction. This alteration could lead to tissue damage. Another factor may involve the tissue’s response to the repair process. During the initial expansion phase, the tissue maintains a high degree of rigidity. The subsequent expansion of the tissue results in a slight tear. This tearing was observed under the microscope in the present study.

Gordon’s solution demonstrated the best performance of the three repair modalities tested in this study, exhibiting the most intact tissue morphology and cellular nuclei. However, it also required the longest duration among the three. Furthermore, microscopic examination following rehydration revealed optimal tissue preservation, with no occurrence of re-solidification observed with Sandison’s solution, nor the gaps seen with Formol-Glycerol solution. These results indicate that among the three repair solutions tested, Gordon’s solution is the optimal choice for the restoration of desiccated hepatic tissues. Although Gordon’s solution necessitates a more extended duration of rehydration, it yielded superior repair and staining outcomes compared to the other two rehydration solutions.

Although all three repair solutions restored nuclear morphology, the air-drying process caused stromal compression and collapse of luminal architecture in the biliary regions of the liver. After repair, we observed areas suggestive of bile ducts and biliary epithelium; however, these structures remained tightly collapsed and closely apposed, precluding definitive identification by H&E staining alone. These observations indicate that the repair solutions have limited efficacy in restoring ductal lumina. Moreover, this study used only H&E staining and did not evaluate prolonged immersion in Gordon’s solution; future studies should systematically assess extended immersion times and supplement H&E with specialized histochemical stains (*e.g.*, PAS, van Gieson, Masson) and immunohistochemical markers to more comprehensively evaluate the effects of rehydration on liver tissue.

Tissue dehydration is a critical step in tissue processing. Tissue-related complications, such as drying up and fragmentation of the specimen, resulting from factors such as equipment malfunction, can impede accurate and timely medical diagnoses. It is essential to recognize that none of the proposed repair methods can restore desiccated tissues to their original conditions. Therefore, early warning mechanisms are essential to prevent potential tissue damage. Given the inherent value of tissue specimens, comprehensive monitoring of the dehydration processor is necessary to ensure its optimal function.

These monitoring measures cover a wide range of parameters, including operational aspects, reagents, procedural nuances, and personnel management. Such measures are essential to mitigate potential adverse impacts and ensuring the safety and integrity of the tissue specimens. The advent of Internet of Things (IoT) technology has made it possible to incorporate remote alarm systems into machinery, thereby ensuring the safety of tissue dehydration and preventing adverse events. In the event of a malfunction, the system can transmit a real-time alert. It is also important to ensure that the remote monitoring and fault remote alarm device of the dehydrator has been installed to ensure the safety of the dehydration process.

## Conclusions

In this study, we found that all three repair solutions exhibited restorative effects on desiccated tissues, but their efficacy varied, along with distinct microscopic appearances. Regarding repair duration, the 24-hour immersion significantly outperformed the 12-hour immersion. Among the three solutions, Gordon’s solution after 24-hour immersion exhibited the best performance, with its microscopic morphology resembling that of normal tissue. Although Formol-Glycerol solution and Sandison’s solution also showed restorative properties, they still displayed notable differences compared to the normal morphology. This study was limited to liver tissue, used only hematoxylin and eosin (H&E) staining, specifically the hepatocytes within it, and did not investigate other staining or other tissue types or extend the observation period to further assess restorative effects. These aspects will be explored in future studies.

##  Supplemental Information

10.7717/peerj.21363/supp-1 Supplemental Information 1 Raw data

## Additional Information and Declarations

### Data Availability

The following information was supplied regarding data availability: The raw data is available in the Supplemental File.
